# Sports Performance Tests for Amputee Football Players: A Scoping Review

**DOI:** 10.3390/ijerph19074386

**Published:** 2022-04-06

**Authors:** Agnieszka Magdalena Nowak, Jolanta Marszalek, Bartosz Molik

**Affiliations:** Faculty of Rehabilitation, Jozef Pilsudski University of Physical Education in Warsaw, 00-968 Warsaw, Poland; jolanta.marszalek@awf.edu.pl (J.M.); bartosz.molik@awf.edu.pl (B.M.)

**Keywords:** field-based tests, amputee soccer, assessment, disability, impairment, athletes, adapted sport

## Abstract

Background: This scoping review aims to identify sports performance tests for amputee football players and to critically analyze the methodological quality, validation data, reliability, and standardization of sport-specific tests to indicate the best-fitting tests. Methods: Electronic database searches were conducted between January 2019 and October 2021. Twelve articles met the inclusion criteria. Qualitative assessment of each study was conducted by STROBE checklist. Results: Twenty-nine sports performance tests were identified. No sports performance test fully met all three criteria associated with the qualitative assessment of tests. The critical appraisal of the articles demonstrates a gap in study design, settings, and main results description. Some inconsistencies were found in the methodological descriptions of tests assessing the same motor skill. A STROBE score of 13 points was considered a satisfactory score for the article (it was obtained by 8 of the 12 studies). The weakest point of the analyzed studies was the description of how the test group size was accessed and later obtained. Conclusions: No test was found that was simultaneously presented as valid, reliable, and standardized. The authors can recommend the use of the two-sports performance tests that are the closest to ideal: the L test and the YYIRT1.

## 1. Introduction

Amputee football (amputee soccer; AF) is an impairment-specific football for people with an amputation or limb deficiency (US Soccer Federation). The major part of AF rules is based on regular soccer rules, while the few paragraphs consider the physical impairment of players [1]. Accordingly, two halves are being played (2 × 25 min) on a smaller pitch (from 60 × 30 to 70 × 55 m) by seven players (six field players, one goalkeeper). Single-leg amputees (either above or below the knee) play without prosthesis on aluminum wrist crutches (field players). Goalkeepers must be single-arm amputees [2]. AF is still developing and has become a point of interest for many researchers since it is a non-Paralympic sport discipline that is applying to enter the Paralympic Games. AF has become greatly popular as a recreational and elite sport. It is also recommended as a continuation of the rehabilitation process for amputees to improve the level of functional fitness, as well as a form of physical activity that allows people to realize themselves as athletes. What is more, AF has a positive impact on body composition and quality of life, and it gives a sense of belonging to society [3,4,5].

It is assumed that AF is classified as a high-intermittent sport with periods of high-intensity activity [6,7]. AF requires from its players a high level of many physical attributes, such as power, speed, strength, balance, agility as well as endurance [8,9]. Short bursts of high-intensity power production and aerobic capacity play a major role in AF performance [2,9]. Some studies have confirmed this, indicating that athletes spend the majority of their playing time in a heart rate zone above 80% of their maximum heart rate (HRmax) [6,7]. Given the high intensity of the game, it is important to emphasize that AF players should be in excellent condition to easily perform the entire spectrum of activities with and without the ball and while moving on crutches [9]. Studies underline the fact that using crutches is quite exhausting for AF players [10]. Therefore, it can be assumed that players should not only be well prepared technically and tactically but also, most importantly, physically for the game, as is the case with able-bodied soccer players [11]. Coaches should be obliged to evaluate the motor performance of players to notice progress or weaknesses in the training process.

In the literature, many different sports performance tests have been reported [2,3,9,12,13,14,15,16,17,18,19,20]. Moreover, by reviewing the literature and observing the various nomenclature of motor abilities used and the different descriptions of the same tests, we decided to organize the sports performance tests for assessing the motor performance of amputee football players to make them transparent and understandable for researchers in the field, coaches, and people interested in this type of sport. Considering how important the periodic assessment of athletes’ motor performance is to both sport-specific and non-sport-specific tests related to AF, the fundamental aim of this study is to identify sports performance tests for amputee football players in a literature review and to critically analyze the methodological quality, validation data, reliability, and standardization of sport-specific tests to indicate the best-fitting tests. Furthermore, the quality of the reviewed articles is checked to indicate the quality of the studies’ descriptions.

## 2. Materials and Methods

### 2.1. Search Strategy, Study Selection, and Data Extraction

Reporting of this scoping review was guided by the Preferred Reporting Items for Systematic Review and Meta-Analysis (PRISMA) statement standards. The review protocol was registered with PROSPERO (CRD42021286911), and the review itself was conducted in January–October 2019 with no restrictions on the date of study publication. It was then regularly updated until November 2021. Electronic databases (EBSCO (SPORTDiscus with Full Text, Academic Search Ultimate, Teacher Reference Center, Health Source: Nursing/Academic Edition, MasterFILE Premier), Web of Science, and PubMed (Medline)) were searched. Database settings were customized for each database (option to search all fields, scientific journals, peer-reviewed articles). The keywords used in the search were divided into three groups: amputee OR amputation AND physical AND soccer OR football and were conducted by the Boolean AND/OR. More specific keywords were not needed due to the small number of publications in the field. The keyword combinations were used according to the databases’ capabilities and were presented in an online repository.

**Figure 1:**
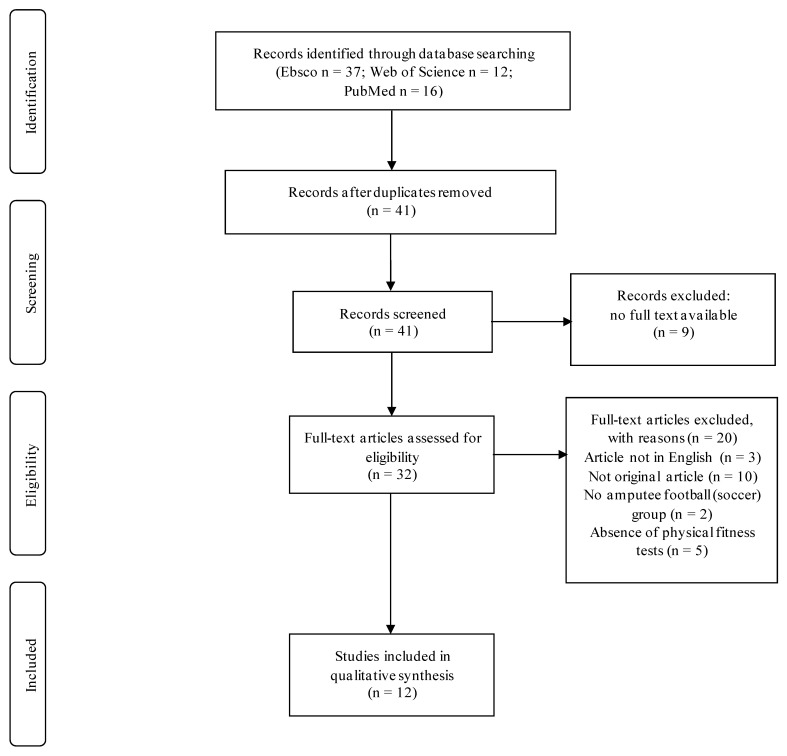
Study selection flow diagram.

In the examination process, the title and the abstract were first checked for compatibility with at least one keyword. If an article met the inclusion criteria, it was carefully selected for this review by making sure that it: was available in an online database in full text (1), was written in English (2), was an original study (cohort, case–control, cross-sectional) (3), involved amputee football players (4), and used sports performance tests as research tools (5). The criteria according to which an article could not be included in the examination were as follows: no keyword in the title and/or abstract, papers of other types (reviews, case reports, conference reports, chapters in books), written in a language other than English, not related to amputee football players, and did not include sports performance tests. We used Microsoft Excel to collect the data and uploaded them to an online repository. The PRISMA flowchart was used to describe the review process (Figure 1). Two researchers (A.M.N., J.M.) independently conducted the process.

### 2.2. Studies Description

First, the studies included in the review are described in a table pointing out the type of research conducted, the purpose of the study, and the characteristics of the study group. A summary description of the included studies is presented in Table 1.

### 2.3. Sports Performance Tests Description

The sports performance tests identified in the literature were analyzed in terms of the type of test and the entire procedure for conducting the test, including athlete preparation, warm-up, how to do the test, number of repetitions, intervals between repetitions of the test or between tests, and variables that are test results. The methodology of the identified sports performance tests is described in Table 2.

### 2.4. Sports Performance Tests’ Quality Assessment

In this phase, we divided the sports performance tests according to their characteristics (motor abilities), which assess balance, aerobic capacity, strength, endurance, power, sprint performance, agility, and flexibility. Two researchers (A.M.N., J.M.) independently assessed all the papers and then consulted the results among themselves.

All found sports performance tests were analyzed for reliability, validity, and standardization based on the authors’ descriptions in the methods section of the articles. Test reliability and validity were recognized based on information about the reliability and validity of the test in the study and whether test references or expert validity were used. Expert validity implies that the researcher, based on their knowledge and experience, selected a sports performance test to assess specific motor abilities, e.g., the 30 m sprint test was used to assess sprint performance. Standardization means that the researchers have written down all the information necessary to repeat the test (participant preparation, environment, methodology, number of repetitions, intervals, outcomes). A description of this assessment is provided below, and points were allocated for each parameter:

- validity, reliability and/or standardization information present and/or cited references that have confirmed validity, reliability and/or standardization and/or expert validity: “1”;
- cited references present but not available or in a language other than English or in unavailable books; no information on validity, reliability and/or standardization or insufficient standardization: “0”.

Table 3 presents the qualitative assessment of the sports performance tests identified through the literature review process.

### 2.5. Studies’ Quality Assessment

The studies included in the review were qualitatively assessed to highlight the value of the papers in terms of their methodological design. To accomplish this, the Strengthening the Reporting of Observational Studies in Epidemiology (STROBE) statement was used, which was created to improve the quality of reported observational studies, and such studies were included in our study. The STROBE statement allows the strengths and weaknesses of the observational studies to be identified and provides an opportunity to generalize the results of the report [21]. The STROBE checklist consists of a checklist of 22 items that relate to the title and abstract (1 item), introduction (2 items), methods (9 items), results (5 items), discussion section (4 items), and other information (1 item) in the articles. One point for each item in the paper was given [22]. Some of these items originally had sub-items. In that case, one point was awarded for more positive responses. The outcome was the score obtained when consensus was reached (A.M.N., J.M.). Discrepancies were resolved by consensus with a third researcher (B.M.).

## 3. Results

Twenty-nine sports performance tests were found in the 12 included studies to assess AF players. They assessed motor abilities such as balance, anaerobic performance (strength, power, sprint performance), aerobic performance (capacity), flexibility, and agility (speed performance) (see Table 3). Despite measuring the same motor ability, the identified tests had different methodologies. For example, the jump test was performed once with and once without a prosthesis, and, in the second case, there was no information about it.

Through 29 sports performance tests, no test met all three quality assessment criteria. In eight cases, participants performed tests with a prosthesis, as marked with an asterisk and presented in Table 3.

### 3.1. Qualitative Assessment of Sports Performance Tests

#### 3.1.1. Reliability

Five out of twenty-nine tests had confirmed reliability in the cited publications (handgrip test, CMJ, L test, YYIRT1, and Berg Balance Scale [2,3,16,19]), and the L test was tested for reliability among amputees.

Two tests, despite the references provided (isometric test of back extensors and trunk flexor test), were not described as reliable or used in the cited publications [3].

#### 3.1.2. Validity

In total, 18 sports performance tests were considered valid based on expert validity and 11 on literature reference; 4 of the 11 cited books were not available.

#### 3.1.3. Standardization

Although all the identified tests had a description of the procedure, only 28% of them met the standardization criteria. Sports performance tests that had complete instructions (subject preparation, environment, methodology, number of repetition, intervals, outcomes) were the T-square test, modified Thomas test, sit-and-reach test, vertical jump test, static balance test, and dynamic balance test [3,9,12,16,20]; 8 tests lacked information about participants’ preparation, 9 tests lacked information about the warm-up, 16 tests lacked information about the number of test repetitions, and 6 tests lacked information about intervals between test attempts. The qualitative assessment of sports performance tests is presented in Table 3.

### 3.2. Qualitative Assessment of Articles

In this scoping review, we included observational studies available in the field of amputee football (5 case–control studies, 6 cohort studies, and 1 cross-sectional study). In total, 10 out of 12 articles met eligibility criteria and were from the past 10 years; 50% of the studies had a study group and a control group. Participants were AF players aged 24–32 years from local [2,9,12,15] or national teams [3,16,17,18,19,20]. Training experience ranged from two months to eight or more years. Two studies did not provide information on players’ training experience [12,14] (see Table 1 for details).

The qualitative assessment of the studies resulted in STROBE scores ranging from 5 to 17 (mean 12.9 points; 65%). Two studies had the highest score of 17 points [17,18], while two different studies had the lowest possible score [14,19]. Six of the twelve studies had an appropriately constructed abstract and title, with two studies indicating the study design in the title or abstract and four studies indicating the study design in the methods section. All studies stated specific objectives, and 11 of 12 studies sufficiently explained the background of the study. In most cases, the participant description was correct. Simim et al. (2018) obtained the highest and the maximum score in the methods section. Providing information on how the study size was obtained in the methods section was the weakest aspect of the evaluation (only 2 of 12 authors reported this data [12,18]). In the results section, two articles met the requirements of item 13 (participants), eight articles met the requirements of item 14 (descriptive data), six articles met the requirements of item 15 (outcome data), three articles met the requirements of item 16 (main results), and six articles met requirements of item 17 (other analysis). In summary, in four studies, the key results concerning the study objectives were presented in the discussion section [3,14,15,20]. The items on limitations, interpretation, and generalizability were met by most of the included studies. Four studies provided information on the source of funding (item 22). The qualitative assessment of the included studies is presented in Table 4.

## 4. Discussion

The purpose of this scoping review was to identify sports performance tests for amputee football (AF) players in the scientific papers and to critically analyze these tests for reliability (i), validity (ii), and standardization (iii) to indicate the best-fitting tests. Along this line, 29 sports performance tests used in AF were found in the current literature (12 studies). We found no sports performance test that would fully meet all three criteria associated with a qualitative assessment of sports performance tests.

When discussing the first parameter (i), the authors of the included studies did not conduct a test reliability examination. The reliability of five tests (YYIRT1, L test, handgrip test, CMJ, and Berg Balance Scale) has been confirmed by the authors of the included studies based on the references [2,3,16,19]. The reliability of only one test, the L test, was verified on amputees, which is an advantage of the reported study [3,23] compared to other tests in which reliability was verified on able-bodied individuals. The authors of the analyzed studies used reliable tools to assess muscle strength [19], lower limb power [17], aerobic capacity [16], and balance [2,3].

The PUT, the isometric back extension test, and the isometric trunk flexion test had inappropriate references to prove the reliability of these tests because the works cited were off-topic [3,17,18]. Consequently, we suggest that researchers and coaches pay attention to the reliability of sports performance tests applied to their groups of athletes.

In terms of validity (ii), from one point of view, the indispensable information was obtained in seven sports performance tests, which included the static balance one-leg test, dynamic balance test, handgrip test, L test, F8W test, YYIRT1, and Berg Balance Scale [2,3,16,19]. Whereas, in the case of four tests, such as the modified Thomas test, the sit-and-reach test, the vertical jump test by the Lewis formula, and the isometric back extension test, we could not approve their validity due to the inability to find the reference cited by the authors [3]. Additionally, about the PUT, it was performed differently than reported in the original paper [24]. Consecutively, it also remains unknown whether the presented PUT is truly valid [16]. Moreover, in articles that used static (Kistler force platform) and dynamic balance tests, isokinetic trunk strength tests, PUT, isotonic sit-ups tests, isometric back extension and trunk flections tests, CMJ and SJ (force plate Sport Expert TM), MBT, CPX two-armed exercise tests, modified Thomas tests, sit-and-reach tests, T-square, and sprint tests, there was no information on validity and reliability verification [2,3,9,12,13,14,16,17,18,19]. It is probably the case that the authors of included studies, when selecting tests to assess the motor abilities of AF players, verified these tests based on their experience and general knowledge (e.g., sprint tests to assess speed or sprint performance); therefore, we decided to give them one point as an expert validation.

It must be admitted that in most sports performance tests, the standardization (iii) was clearly explained. Information regarding the starting and finishing positions, the number of repetitions and break times, and the type of movement (running, walking with or without prosthesis) was adequately introduced. This renders them easily repeatable and, thus, helpful for both researchers and coaches. When analyzed in detail, 8 of the 29 test descriptions met all standardization criteria (T-square test, modified Thomas test, sit-and-reach test, vertical jump test, static balance test, and dynamic balance test [3,9,12,16,20]). For a test such as the YYIRT1, the only information about the number of repetitions of the test performed was missing, but we believe that this information is not necessary in this case because this type of aerobic capacity test is usually performed only once due to the maximal stimulation of the aerobic system, after which a long recovery is necessary [25]. The lack of descriptions regarding the warm-up and intervals between repetitions in sprint tests [9,14,19], in which a maximal effort is performed, deserves significant criticism since all these elements are crucial in the assessment of anaerobic performance. In the case of balance tests, information about the use of a familiarization session is important in the context of repeating and comparing the test in the future, and the question of whether and how this session affects test performance (learning process) and the final result is still unknown [26].

On the other hand, some of the tests might be misleading, e.g., the PUT, sprint tests, CMJ (by Myotest), MBT, and CPX two-armed exercise tests, in which it was not explained why and how the procedures were followed and how they were adapted for amputees [9,13,16,17,18]. The PUT did not have information about the position and the type of movement included in the description, as well as whether a prosthesis was used during this test and other tests [13,17,18]. Because of these confusions, we expected that performing the MBT in a seated position with or without a prosthesis might influence the stability of the trunk position, and, consequently, the final results might be different (athlete sits close to the wall vs. athlete performs a full backward and forward movement to complete the task). In some locomotion tests, participants used a prosthesis (L test and F8W test), while in others, they performed the tests on crutches without a prosthesis (T-square). At this point, it is worth asking ourselves under which conditions we want to evaluate the AF players, as it must be remembered that the athlete is moving on crutches during the match. The same dilemma regarding the use of a prosthesis or not has been noted in vertical jump tests [3,9] and balance tests [2,12]. Consequently, the reader does not know if these tests were performed in a single-leg standing position or if the athletes had three or four points of support. Moreover, we noted several discrepancies concerning the start of the tests. For the T10, T20, and T30 procedures [9], there was no information about the starting position or whether the starting signal was given by the researcher or whether the athlete decided to start the test. Then, in the MBT, it was not clear where the starting point was for measurement. Without such information, it is difficult to compare the results obtained by different groups of participants and then repeat and compare the tests with each other. The differences in results are likely due to erroneous measurements rather than the athletes’ skills, making the ratings unreliable. Therefore, it is recommended that in future papers, authors describe their tests accurately.

The studies included in this review have many limitations in the clarity of the names of the motor abilities assessed in sports performance tests because of various wording. In other words, three different groups of researchers used different terms to match tests to the physical attribute; for instance, T30 was used to assess anaerobic performance, sprint performance, or movement speed [9,16,19]. It would be clearer for readers to use only one term. Surprisingly, the L test and the F8W have been classified as sprint tests, together with the T10, T20, and T30, which are speed tests [3,14,16,19]. It becomes obvious that the sprint tests were performed as fast as possible in a straight line, while the L test and the F8W were performed with changes in direction, which may affect the change in running speed and is more to assess agility than speed. Moreover, the result of the L test and the F8W may consist of the route execution technique, which is unlikely for the sprint tests.

A similar observation was made for the vertical jump tests and the MBT. The latter has been used as a power test, a muscle test, a neuromuscular performance test, and an anaerobic performance test and has been positioned as a test focused on strength assessment [3,9,17,18]. Given these achievements, we suggest classifying the MBT as a power assessment because it is the same physical attribute that vertical jump tests assess. We believe that future manuscripts should pay more attention to the terms and expressions used in the sports performance tests and to the description of the physical attributes. Maintaining this level of vocabulary clarity will be beneficial to both coaches and athletes in understanding which motor abilities are being tested in each sports performance test.

The articles included in this review had large discrepancies in scoring in the qualitative assessment. The authors of the current study believe that the methods and results sections of the included studies need the most correction and attention. First, providing the study design in the abstract and/or methods section is important because it gives the reader an understanding of what type of research they will be dealing with. Most studies correctly described the participants. The reader can read about: eligibility criteria and how participants were selected, outcomes, exposures, predictors, potential confounders, and details of assessment methods. The above-mentioned description is important because it indicates whether the study group was homogeneous and whether there were confounding factors.

In this review, the authors of the included studies did not mention any possible confounding factors or description of the test location (whether the tests were performed in the same setting, such as a gym, laboratory, or outdoor soccer pitch). Different conditions and environments can affect the results: e.g., headwind, a slippery floor in sprint tests, and low temperatures can cause poorer results in sprint or flexibility tests. In addition, researchers and coaches should be cautious when interpreting their results concerning the already existing results of others, as there have been times when the results of the same test have depended on different variables. For example, in the PUT, the duration of the test or the number of repetitions performed within a specified time was evaluated; in the sprint tests, the time or speed of the distance covered was evaluated; in the jump tests (vertical jump, CMJ, SJ), the height of the jump or power was evaluated.

What is more, we were concerned about the lack of explanation of how the study group size was obtained (only two articles reported this [12,18]). This issue is particularly relevant when judging null results, which might indicate that there was no real difference between the study groups or that the power of the statistical analysis was too low to detect a real difference. It is worth noting that some studies on AF players included relatively few participants (6–33 people). In the result section, the items were quite complex, and a study had to meet most of the criteria for each item to receive one full point. If a sub-criterion did not apply to the study in some cases, we did not count it. It seems worrying that most articles do not state the key results at the beginning of the discussion section (item 18). Another important point to indicate is if the purpose of the study was achieved in order to lead the discussion section fluently.

Although the STROBE checklist was designed for observational studies, it is important to keep in mind when using this tool that not all criteria are mandatory for every subtype of study, e.g., cohort studies usually do not have any follow-ups or reduction in the number of participants because they have only one group and the study is conducted over one or two days. The STROBE statement is a particularly detailed tool; on one hand, it can help in the preparation of the manuscript, but, on the other hand, it can cause difficulties in the evaluation of the study due to its precision. Considering the presented conclusions and the fact that most of the studies were single-case studies and that we could not give a positive score for some criteria (not because there was an error in the article but because the criteria did not apply to the study), we judged that 60% (13 points) was a satisfactory score, and, thus, 8 of the 12 articles achieved it.

### Limitations and Perspectives

This is the first review to bring together all the sports performance tests used in AF and organize them in detail in terms of motor abilities and test descriptions. The available literature lacks a “gold standard”, a battery of sports performance tests, or a compilation of which tests are dedicated to AF players (sport-specific tests). Our study indicates that some tests, based on their standardization, may be suitable for assessing sports performance in AF, and coaches may use them in their practice. However, further research is needed to investigate the tests’ validity and reliability and characterize them for AF players.

We understand that the literature search performed in this research field may be conducted differently in future studies. This manuscript presents a structured way of literature review (keywords, inclusion/exclusion criteria). Other authors may search the literature using different methodology and other guidelines for reporting the main types of studies, such as the STROBE guidelines that were used in our study. However, in our opinion, recommendations for future studies seeking sports performance tests in a specific sport should be structured as a research review and a presentation of the advantages and disadvantages of the tests and research, such as was done in this study (quality of paper, presence of validity and reliability of tests, and the completeness of description of selected tests). A well-planned research review and manuscript organization will be important for the next steps in AF development as a future Paralympic sport, considering the development of sports classification based on evidence (evidence-based classification system) [27]. The International Paralympic Committee (IPC) has outlined the steps in this process, and the identification of tests in this manuscript is relevant to step 2 (identifying key activities and determinants) and step 3 (identifying appropriate tests to assess key determinants) of the IPC classification process [28]. Authors of future research may consider this rationale and address the need for an evidence-based classification approach as a purpose of their work.

## 5. Conclusions

Our study constitutes a practical and detailed description of the sports performance tests identified in the literature and includes a qualitative assessment of sports performance tests and a qualitative evaluation of the included articles. The authors of the studies included in this review have verified the reliability and validity of sports performance tests based on results from others’ studies. Considering the final conclusions of the reviewed studies and our evaluation of these studies, we conclude that none of the 29 tests from the 12 research papers included in this review were simultaneously reported as valid, reliable, and standardized. We found few tests for amputee football players, which were only partially verified for validity and reliability; thus, we recommend verifying those tests using, for instance, the test–retest method [29]. Despite the deficiencies in the test descriptions, we recommend using two sports performance tests: the L test and the YYIRT1, to assess agility and endurance, respectively.

## Figures and Tables

**Table 1:** General description of included studies (*n* = 12; studies arranged in chronological order).

Authors	Type of Study	Purpose	SG / CG	Training Experience of SG
Buckley et al., 2002	CC	To determine the balance performance of active lower limb amputees during quiet standing and under dynamic conditions.	*n* = 6 (AF) 25.7 ± 5.8 yrs. / *n* = 6 (AB) 24.7 ± 2.7 yrs.	ND
Yazicioglu et al., 2007	CS-C	To investigate the effect of playing football on balance, muscle strength, locomotor capabilities, and health-related quality of life in subjects with unilateral below-knee amputation.	*n* = 12 AF, 28.3 ± 4.6 yrs. / *n* =12 (AMP) 29.8 ± 1.4 yrs.	≥6 mths
Ozkan et al., 2012	C	To investigate the relationship between body composition, anaerobic performance, and sprint performance of AF.	*n* = 15 (AF) 25.5 ± 5.8 yrs.	3.3 ± 2.9 yrs.
Simim et al., 2013	C	To describe anthropometric and physical characteristics of AF and to compare these results, taking into consideration the players’ tactical function, and to verify if there are differences between HR after maximum test and the employment of six equations for prediction of HRmax.	*n* = 12 (AF) 29.3 ± 8.6 yrs.	≥5 yrs.
Mine et al., 2014	C	To examine relationships between quickness and speed performance in AF.	*n* = 10 (AF) 25.8 ± 4.32 yrs.	ND
Wieczorek et al., 2015	C	To find the relationship between handgrip strength and sprint time in AF.	*n* = 13 (AF) 26.1 ± 7.7 yrs.	30.8 ± 14.3 mths
Guchan et al., 2017	CC	To determine the effects of playing soccer on various components of physical performance such as body composition, muscular endurance, anaerobic power, flexibility, balance, and speed of individuals with transtibial amputation.	*n* = 12 (AF) 26.67 ± 7.76 yrs. / *n* = 12 (AMP, sedentary) 33 ± 6.7 yrs.	≥1 yr.
Simim et al., 2017	CC	To quantify the degree of game-induced muscular fatigue in AF.	*n* = 33 (AF) 31 ± 7 yrs. / *n* = 5 (AF, not playing all matches)	≥4 yrs.
Simim et al., 2018	C	To investigate the match demands of amputee football and its impact on muscular endurance and power.	*n* = 16 (AF) 32 ± 5 yrs.	≥5 yrs.
Mikami et al., 2018	CC	To examine the difference in measured CPX values among two-legged, one-legged, and two-armed exercises in AB, and to preliminarily evaluate the endurance of AF through CPX with two-armed exercise.	*n* = 20 (AB) 28.3 ± 5.6 yrs. / *n* = 8 (AF) 36.4 ± 5.7 yrs.	0.4–5 yrs.
Miyamoto et al., 2019	C	To analyze sprint motion in outfield positioned AF using crutches and to clarify the relationship between sprint speed and sprint motion.	*n* = 12 (AF) 42.3 ± 4.6 yrs.	3.58 ± 2.48 yrs.
Zwierko et al., 2020	CC	To examine postural control during single-leg stance test with progressively increased balance-task difficulty in soccer players with unilateral transfemoral amputation compared to AB soccer players.	*n* = 11 (AF) 27.45 ± 5.2 yrs. / *n* = 11 (AB football players) 21.91 ± 3.11 yrs.	8.27 ± 3.63 yrs.

AB—able-bodied; AF—amputee football players; AMP—individuals with amputation; C—cohort; CC—case–control; CG—control group; CS—cross-sectional; CS-C—cross-sectional control; mths—months; ND—no data; SG—study group; yrs.—years.

**Table 2:** A detailed description of the sports performance tests in included studies.

I	II	III					IV
Authors	Methods	Test Descriptions					Outcomes
		Participants’ Preparation	Warm-Up	Procedures	Repetitions	Breaks	
Buckley et al., 2002	Static balance test (Kistler force platform)	sportswear; prosthesis	ND	comfortable position on the force platform surface with feet equidistant from a central dividing line, hands on the hips; a large visual target at eye level on a wall 5 m from the force platform; stand stationary, look straight at the visual target for 30 s	ND	ND	CP excursion range, sum of the square’s deviations from the mean CP location in the AP and ML directions
	Dynamic balance test (modified dynamic stabilimeter)		approx. 5 min; standing on the stabilimeter	ML—standing on the board pivoted side-to-side in the frontal plane; AP—pivoted forwards and backwards in the sagittal plane; trials in a random order; place hands on hips, focus on a large visual target positioned at eye level on a wall 5 m in front of them	3 trials of 20 s	step down from the stabilimeter	time spent in and out of balance; number of times the board contacted the ground; mean number of contacts per limb (prosthetic or intact, dominant, or non-dominant) or per direction (forwards or backwards)
Yazicioglu et al., 2007	Berg Balance Scale	ND	ND	14 tasks common in everyday life (sitting, standing, reaching, leaning over, turning, and looking over each shoulder, turning in a complete circle, and stepping)	ND	ND	each item is scored on a scale from 0 to 4, max. 56 points
	One-leg static balance test (KAT 2000; Kinesthetic Ability Trainer; Breg, Vista, CA)	ND	5 min.	standing on the intact limb; arms folded across the chest, the knee kept in approx. 10 degrees in flexion; unable to maintain a balance and touch the railing—test discontinued and restart performed in the first difficulty level according to subject’s body mass	once a day, for 3 days	15 min.	distance from the central point to the reference position of each trial; balance index
	Dynamic balance test (KAT 2000; Kinesthetic Ability Trainer; Breg, Vista, CA)	prosthesis		standing on both legs; as above			
	Isokinetic muscle strength test (Cybex dynamometer)	ND	ND	peak torques of nonamputee side knee in extension and flexion; perform maximal concentric-concentric motion at angular velocities of 60, 120, and 180 degrees/s	once	20 s	Nm
Özkan et al., 2012	CMJ, SJ (force plate; Sport Expert TM, MPS-501 multi-purpose measuring system, Tumer Electronic LDT, Turkey; centimeter)	no crutches; no prosthesis	Test familiarization	jump as high as possible; SJ: starting position with knees flexed to 90°, hands fixed on the hips, and no allowance for preparatory counter movement; CMJ: performed from an upright standing position, hands fixed on the hips, and with a counter movement preparatory phase, with end position as SJ starting position	3 CMJs; 3 SJs	2 min.	jump height; total work produced in each jump (the Genuario and Dolgener formulas)
	T10, T20, T30 (light gates with timing system; Prosport, Tumer Electronics, Ankara, Turkey)	crutches; no prosthesis	ND	indoor court; light gates placed at the start and at the finish of each sprint test	2 times each distance	1 min.	time
Simim et al., 2013	T20 (stopwatch)	crutches	10 min, test familiarization	official field with natural grass; player initiated a movement	ND	at least 24 h of recovery between testing sessions; 5 min. between the two first tests on day one; 3 min. rest between each T-square trial	time; mean speed
	T-square (stopwatch)				3 times		time
	The YYIRT1; Polar F5 to HR_max_; Six equations to predict HR_max_			official field with natural grass; 20 m shuttle run test with increasing velocities, 10 s of active recovery between runs until exhaustion; test end if: participant fails twice to reach the front line within the time limit or is unable to complete another run at the imposed speed; HR_max_ record immediately after the test	ND		total distance; HR_max_ result compared with 6 equations used to predict HR_max_
Mine et al., 2014	T30 (electronic timing system)	rubber-soled track shoes	ND	photocells set on 0, 5, 30 m; standardized starting position; players started the approx. 30 cm back from the starting line; quickness on 5 m, speed on 30 m	3 times	3 min. intervals	time
Wieczorek et al., 2015	Handgrip test (SEHAN hydraulic hand dynamometer, Jamar)	ND	ND	sit with arms along the body, elbow joint in 90° flexion, forearm, wrist in a neutral position; grip the handle of the dynamometer	twice by each hand	ND	the highest value
	T30 (Fusion Smart Speed System; Fusion Sport, Coopers Plains, QLD, Australia)	crutches	ND	8 infrared working gates; 3 m distance between photocells and mirrors; splits recorded: 1, 5, 10, 15, 20, 25, 30 m; standing start; deciding themselves when to start; stretched crutches, no crossing the starting line	2 times	ND	time; mean running velocity
Güçhan et al., 2017	Sit-ups isotonic	prosthesis; shoes	5 min.	lie back with bent knees and sit up until the scapula is no longer in contact with the surface	ND	2 min. between tests	repetitions; time
	Isotonic PUT			trunk and head to the floor and push the body up			
	Back extensors isometric			lie on a table face down, with inguinal points and lower body on the end table, upper body over the table; cross arms in front of the shoulders, raise the trunk; assessor fixed participant’s leg			time sustaining in the position
	Trunk flexors			lie back with knees flexed; arms straight toward knees, raise head, neck, shoulders stay in the position			
	Vertical jump test			stand up, fix amputated limb next to the wall, extend arm above; the end of the longest finger was marked before and after a jump; jump vertically; repetition with intact limb near the wall; distance between two marked heights	3 times for both sides		the best result; Lewis’ formula (anaerobic power)
	Modified Thomas test			sit on the end of a table and lie down, with hip joint fixed 28 cm away from the end of the table; flex the contralateral lower limb maximally with arms; tested both sides	ND		distance between the table and the tested knee
	Sit-and-reach test	no prosthesis; no shoes		sit on the floor, knees straight, feet resting vertically; reach forward with straight arms as far as possible; distance between the toes and the longest finger	3 trials		the best result
	Berg Balance Scale	prosthesis; shoes		14 items	ND		each item scored 0–4
	L test			walk an “L” shaped path (7 × 3 m); starting position: sit on a chair without armrests; get up from a chair, walk with usual pace to the end and come back to the sitting position			time
	F8W test			stand in the middle between 2 cones and complete the 8-shaped path with preferred speed walk, come back to the same point; 1.22 × 1.52 m			time; steps
Simim et al., 2017	PUT	ND	tests familiarization; dynamic warm-up and stretching; 1–3 repetitions of each test	max. number of repetitions in 60 s; result divided by body mass	ND	5 min. between tests	repetitions; relative measure
	CMJ (accelerometer Myotest, Sion, Switzerland; centimeter)			use preliminary movement by rapidly flexing the knee, before launching the body vertically	3 trials	as above; 30 s between jumps; 1 min. between throws	jump height; power
	MBT (medicine ball 3 kg)			sit with your back against the wall; lower back stays in contact with the wall during the test; hold a medicine ball with both hands against a chest and throw it on command as far as possible			distance
Simim et al., 2018	PUT	ND	tests familiarization; dynamic warm-up and stretching; 3 repetitions of each test	max. number of repetitions in 60 s; result divided by body mass	ND	one day; pre-and post-match; 5 min. rest between tests	repetitions; relative measure
	CMJ (accelerometer Myotest, Sion, Switzerland)			use preliminary movement by rapidly flexing the knee, before launching the body vertically		as above; 30 s between jumps; 1 min. rest between throws	jump height; power
	MBT (medicine ball 3 kg)			sit with your back against the wall; lower back stays in contact with the wall during the test; hold a medicine ball with both hands against a chest and throw it on command as far as possible			distance
Mikami et al., 2018	CPX exercise test (Strength Ergometer; Strength Ergo 8, Mitsubishi Electric Engineering Co., Ltd., Tokyo, Japan); Expired gas monitoring breath-by-breath (cardiorespiratory exercise monitoring system, AE-310s; Minato Medical Science Co., Tokyo, Japan); Fatigue (Modified Borg Scale)	ND	ND	a multistage, Ramp-wise upgrading continuous load; two-legged exercise: Ramp 25 M/min.; one-legged exercise: used leg on the dominant hand side; Ramp 15 W/min.; two-armed exercise: Ramp 15 W/min.; Modified Borg Scale after exercise	ND	ND	anaerobic threshold value of oxygen uptake/weight; peak value of oxygen uptake/weight; HR; VE; WR; c-RPE; p-RPE
Miyamoto et al., 2019	T30 (electronic timing gates, TC Timing System, Brower Timing System, USA)	crutches; no prosthesis	ND	splits recorded: 10, 20, 30 m; sprint; running style by supporting both crutches together for 2 steps; the 30-m sprint test (10 m intervals); speed (between 10 to 20 m)	2 trials	ND	time
Zwierko et al., 2020	Static balance test with open eyes (Biodex Balance System Inc., Shirley, NY, USA)	barefoot	3 trials of 20 s adaptation (in 12, 8, and 4 level of platform stability)	12 dynamic stability levels (12 is the most stable, 1 is the most unstable); single-leg stance on rigid platform; single-leg stance with decreasing platform stability—levels 8 to 4; single-leg stance with platform stability—level 4; 20 s each balance task; during the tests, look straight ahead with arms folded along the chest	3 trials	10 s	OSI; API; MLI

AP—anteroposterior; API—anterior–posterior index; % BF—relative body fat; BI—balance index; BMI—body mass index; CMJ—countermovement jump; CP—center of pressure; CPX—cardiopulmonary exercise test; F8W—figure-of-8 walk; HR - heart rate; HR_max_—maximum heart rate; HRQOL—health-related quality of life; J-P method—Jackson and Pollock method; MBT—medicine ball throw; ML—mediolateral; MLI—medio-lateral index; ND—no data; OSI—overall stability index; PUT—push up test; RPE—rating of perceived exertion; c-RPE—central rate of perceived exertion; p-RPE—peripheral rate of perceived exertion; SJ—squat jump; T10, T20, T30—10, 20, 30 m sprint test; VE—ventilation equivalent; WR—work rate; YYIRT1—the Yo-Yo intermittent recovery test—level 1.

**Table 3:** Quality assessment of sports performance tests in the review (*n* = 12).

I	II	III	IV		
Physical Attribute Tested	Test Name (and Tools)	Authors	Sports Performance Tests Assessment		
			R	V	S
Balance	Static balance test (Kistler force platform) *	Buckley et al., 2002	0	1	0
	Dynamic balance test (modified dynamic stabilimeter) *		0	1	1
	Static balance test (Biodex)	Zwierko et al., 2020	0	1	1
	One-leg static balance test (KAT 2000)	Yazicioglu et al., 2007	0	1	0
	Dynamic balance test (KAT 2000) *		0	1	1
	Berg Balance Scale	Yazicioglu et al., 2007, Güçhan et al., 2017	1	1	0
Muscle strength	Isokinetic trunk strength test (Cybex dynamometer)	Yazicioglu et al., 2007	0	1	0
	Handgrip test (hydraulic hand dynamometer)	Wieczorek et al., 2015	1	1	0
	PUT	Simim et al., 2017, 2018	0	1	0
	Isotonic PUT	Güçhan et al., 2017	0	1	0
	Isotonic sit-ups test		0	1	0
	Isometric back extension test *		0	1	0
	Isometric trunk flection test		0	1	0
Power	Vertical jump tests—CMJ, CMJs, SJ, SJs (force plate Sport Expert TM)	Özkan et al., 2012	0	1	1
	CMJ (accelerometer Myotest)	Simim et al., 2017, 2018	1	1	0
	MBT (medicine ball 3 kg)		0	1	0
	Vertical jump test (Lewis’ formula) *	Güçhan et al., 2017	0	1	1
Anaerobic performance (sprint and movement speed ^1^)	T10, T20, T30	Özkan et al.2012	0	1	0
	T20	Simim et al., 2013	0	1	0
	T30 (5 m)	Mine et al., 2014	0	1	0
	T30 (1, 5, 10, 15, 20, 25 m)	Wieczorek et al., 2015	0	1	0
	T30 (10, 20 m)	Myiamoto et al., 2019	0	1	0
	L test *	Güçhan et al., 2017	1	1	0
	F8W test *		0	1	0
Aerobic capacity	YYIRT1	Simim et al., 2013	1	1	0
	CPX two-armed exercise	Mikami et al., 2018	0	1	0
Flexibility	Modified Thomas test *	Güçhan et al., 2017	0	1	1
	Sit-and-reach test		0	1	1
Agility	T-square	Simim et al., 2013	0	1	1

V—valid; R—reliable; S—standardization; CMJ—countermovement jump; CPX—cardiopulmonary exercise test; F8W—figure-of-8 walk; MBT—medicine ball throw; PUT—push up test; SJ—squat jump; T10, T20, T30—10, 20, 30 m sprint test; YYIRT1—the Yo-Yo intermittent recovery test—level 1; “1”—presence of validity, reliability, standardization; “0”—absence of validity, reliability, standardization; *—test performed with prosthesis; ^1^—sprint refers to tests in which movement is as fast as possible in one line, movement speed refers to tests in which movement is as fast as possible with changing directions.

**Table 4:** The STROBE qualitative assessment of included studies.

		Introduction		Methods									Results					Discussion				Other Information	STROBE Points ^1^
Items	1	2	3	4	5	6	7	8	9	10	11	12	13	14	15	16	17	18	19	20	21	22	Total
Authors																							
Simim et al., 2018	0	1	1	1	1	1	1	1	1	1	1	1	0	1	1	0	1	0	1	1	1	0	17
Guchan et al., 2017	1	1	1	0	0	1	1	1	1	0	1	1	0	1	1	0	0	1	1	1	1	0	15
Simim et al., 2017	1	1	1	1	1	1	1	1	1	0	1	1	0	0	1	0	1	0	1	1	1	1	17
Wieczorek et al., 2015	0	1	1	0	0	1	0	1	0	0	0	0	0	0	0	0	0	0	0	1	0	0	5
Simim e al. 2013	0	1	1	1	1	1	1	1	1	0	1	1	0	1	0	1	1	0	0	1	1	0	15
Ozkan et al., 2012	0	1	1	0	0	1	1	1	0	0	1	0	1	0	0	0	0	0	0	1	1	0	9
Yazicioglu et al., 2007	1	1	1	1	0	1	1	1	1	0	1	1	1	1	1	0	0	0	1	1	1	0	16
Mikami et al., 2018	0	1	1	0	0	0	1	1	0	0	1	1	0	1	1	0	0	0	1	1	1	1	12
Mine et al., 2014	1	0	1	0	0	0	1	0	0	0	1	0	0	0	0	0	0	1	0	0	0	0	5
Buckley et al., 2002	0	1	1	0	1	1	1	1	1	1	1	1	0	1	0	1	1	0	0	1	1	0	15
Miyamoto et al., 2019	1	1	1	0	0	0	1	1	0	0	1	0	0	1	1	1	1	1	1	1	1	1	15
Zwierko et al., 2020	1	1	1	0	0	0	1	1	0	0	1	1	0	1	0	0	1	1	1	1	1	1	14

^1^—max. 22 items; 1—title, abstract; 2—background; 3—objective; 4—study design; 5—settings; 6—participants; 7—variables; 8—data sources, measurement; 9—bias; 10—study size; 11—quantitative variables; 12—statistical methods; 13—participants; 14—descriptive data; 15—outcome data; 16—main results; 17—other analysis; 18—key results; 19—limitations; 20—interpretations; 21—generalizability; 22—funding.

## Data Availability

The data presented in this study are openly available in FigShare at: https://doi.org/10.6084/m9.figshare.16850194.v1 (accessed on 15 February 2022) and https://doi.org/10.6084/m9.figshare.16850185.v1 (accessed on 15 February 2022).
